# Assessment of Prevalence, Beliefs, and Habits of Hookah Smoking Among People with a Medical Background Compared to People with a Non-medical Background: A Cross-sectional Self-administered Questionnaire-based Study

**DOI:** 10.7759/cureus.735

**Published:** 2016-08-15

**Authors:** Pankaj Gupta, Ashna Jain

**Affiliations:** 1 Department of Conservative Dentistry and Endodontics, Nair Hospital Dental College; 2 Intern, Nair Hospital Dental College

**Keywords:** waterpipe, smoking, medical background

## Abstract

Introduction: Hookah smoking has seen a reemergence in popularity in the last 30 years, particularly in the young urban population. This study aimed to compare the prevalence of and the attitude and beliefs about hookah smoking of people with a medical background and compare it with people from a non-medical background.

Materials and methods: An anonymous questionnaire with ten questions about various aspects of hookah smoking was formulated using Google forms®, which was then circulated via Facebook®, Whatsapp® and emails to the intended participants and all responses were recorded and analyzed.

Results: The total number of respondents were 470. The number of respondents with a medical background was 45.31%. The percentage of the respondents with a medical background who smoked a hookah was 28.63%, while the same percentage of the respondents with a non-medical background was 63.42. The perception of hookah being less harmful than a cigarette was not found to be statistically different between the two groups. Respondents with a medical background were more ignorant of the presence or absence of tobacco in the hookah they smoked. The average duration of the hookah smoking habit, the frequency of its use per month, and the average lengths of the hookah smoking session were 3.52 years (95% CI of 3.21 to 3.82), 1.946 (95% CI 1.799 to 2.093), and 58.90 minutes (95% CI of 54.42 to 63.37), respectively.

Conclusion: The knowledge about the ill effects of smokeless tobacco should be integrated into the structured teaching curriculum of undergraduate medical and dental courses as they prepare future physicians and dental surgeons for an anti-tobacco campaign.

## Introduction

Tobacco use is one of the leading causes of death throughout the world. In 2009, the World Health Organization reported that 12% of all adult deaths could be attributed to tobacco while the same number was 16% in India, 17% in Pakistan, and an astonishing 31% in Bangladesh. In 2004, five million deaths were a direct result of the use of tobacco in smoking or smokeless forms, which translated to one death every six seconds [1]. The primary form of tobacco consumption is through cigarette smoking. Because of many of the anti-smoking campaigns throughout the world, there has been a decline in the prevalence of smoking. Although the prevalence of cigarette smoking has decreased, there has been a marked increase in the prevalence of other forms of tobacco consumption. One of the main alternative methods of tobacco consumption, which has seen an exponential rise in popularity, is the water pipe or the hookah.

Water pipe smoking has its roots in India and Persia, originating about 400 years ago [2]. It is also known by many other names, such as hookah, narghile, arghile, shisha, and hubble-bubble [3]. In its traditional form, it was used mainly by elderly people in the rural areas of South Asian and Mediterranean countries; however, in the last 30 years or so, there has been a phenomenal rise in its use in the urban population, especially among teenagers and young adults [4-6]. This resurgence in the popularity of the water pipe after a gradual decline in the 20^th^ century coincided with the manufacturing of sweetened and flavored tobacco specifically made for water pipe smoking known as "maassel". The easy availability, along with aggressive advertising campaigning on mass media and a myriad of flavors, were enough to cause a massive increase in the popularity of water pipe smoking [7-8]. This trend is worrisome for several reasons; namely, unlike cigarette smoking, there are few regulations and legislation in place regarding the advertising, sale, and smoking of hookah. Secondly, the perception of the hookah being less harmful than a cigarette, coupled with the flavors in which hookah is available, make it socially more acceptable compared to cigarettes. It is not surprising that hookah bars and lounges have mushroomed exponentially in urban areas.

Health care professionals are at the forefront in sensitizing the general population about the harmful effects of smoking tobacco and also play an important role in guiding the patients through the de-addiction process. For this to happen, the health care professionals should be aware of the ill effects of tobacco consumption in all forms. It is also imperative that they have a strong negative attitude towards it, which is possible only when they don’t nurture the habit themselves.

This study was formulated based on the above-mentioned background. This study was designed to evaluate the attitude and habits of health care professionals towards hookah smoking and compare it to participants with a non-medical background.

## Materials and methods

To assess the knowledge, attitude, and habits of young adults (between the ages of 21 to 30 years) with a medical background and compare it with individuals from a non-medical background, an anonymous questionnaire with ten questions regarding various aspects of hookah smoking was formulated using Google forms® (http://docs.google.com/forms). The questionnaire was formulated in collaboration with two experts in the field. Simple random sampling was used for choosing the participants. The link to the survey was circulated to the intended participants via email, Whatsapp®, and hosted on the Facebook® page of the authors. The various questions in the questionnaire were related to the habit, frequency, and duration of hookah smoking and about the individual’s perception about the harmfulness of hookah smoking compared to cigarette smoking. Other questions inquired about hookah smoking being undertaken as a group activity and if the participant had recommended hookah smoking to anyone. Lastly, it was inquired if the participant smoked anything else apart from a hookah. The questionnaire was kept live for a period of six weeks, and the individuals were reminded to fill it up by a weekly email, Whatsapp® message, or Facebook message. Data were analyzed using the Statistical Package for Social Sciences (SPSS) software version 21.0 (SPSS® Inc., Chicago, IL, USA). Numbers and percentages were produced to summarize categorical and nominal data. In addition, the Chi -square (χ2) test was used for comparisons among subgroups. The level of significance was set at (P < 0.05).

Participants who agreed to participate in this study were explained the nature and the objectives of the study, and informed consent was obtained.

## Results

The questionnaire was sent to 500 individuals of which 470 individuals responded (response percentage 94%). Of the 470 respondents, 52.12% were males and 47.87% were females. Of the total respondents, 45.31% had a medical background and the rest (54.68%) had a non-medical background. Of the total respondents, 78.08% thought that cigarettes were more harmful than hookah smoking while 47.65% actually smoked a hookah. An astonishing 66.80% respondents had recommended hookah smoking to others. Out of the total respondents smoking a hookah, 74.10% did not know if the hookah smoked by them contained tobacco, while 17.85% responded positively to the presence of tobacco in their hookah and 8.03% responded positively to the absence of tobacco in hookah they smoked. The average duration of smoking was 3.52 years (95% CI of 3.21 to 3.82 years) while the average frequency of hookah smoking was 1.946 (95% CI 1.799 to 2.093) times per month. The average time of a hookah smoking session was 58.90 minutes (95% CI of 54.42 to 63.37). Hookah smoking was performed as a group activity (97.32%) in the majority of the time reported. More than half the respondents (58.03%) smoked other things in addition to the hookah.

The results of the survey are summarized in Table 1.

**Table 1:** Summary of Responses

Summary of Responses	
1. Total number of respondents	470
2. Response rate	94%
3. Gender	
Male	52.12%
Female	47.87%
4. a. Respondents with a medical background	45.31%
b. Respondents with a non-medical background	54.68%
5. a. Respondents who think cigarettes are more harmful	78.08%
b. Respondents who think hookah smoking is more harmful	21.91%
6. a. Respondents smoking hookah	47.65%
7. Does the hookah smoked contain tobacco?	
a. Yes	17.85%
b. No	8.03%
c. Don’t know	74.10%
8. Mean duration of hookah smoking habit in years	3.52 years
9. Mean frequency of hookah smoking per month	1.946 per month
10. Mean duration of hookah smoking session	58.90 minutes
11. Do you share the hookah or smoke alone?	
a. Share	97.32%
b. Alone	2.67%
12. Smoking other stuff apart from a hookah	
a. Yes	58.03%
b. No	41.96%

## Discussion

The purpose of the study was to compare the prevalence, knowledge, awareness, and hookah smoking habits of individuals with a medical background with those from the non-medical background. The need for the study was to find out if individuals with a medical background were themselves sensitized to the ill effects of hookah smoking and if they carried a strong negative attitude towards hookah smoking, which could be concluded from them not taking up the habit themselves. Lastly, the study was also conducted to determine if the respondents with a medical background harbored the widespread belief of the hookah being less harmful than the cigarette.

The prevalence of hookah smoking in the present study was found to be 47.65%, which is higher than the prevalence reported by Akl, et al. in a meta-analysis of 38 studies on hookah smoking [9]. This may be explained in part due to the age group from which the respondents were chosen in the present study (range: 21-30 years).

In the present study, 50.61% males and 44.44% females smoked a hookah. This pattern of higher hookah smoking prevalence in males is consistent with other studies [6, 10].

The high prevalence of hookah smoking among females, when compared to cigarette smoking, is likely because, unlike cigarette smoking, water pipe smoking does not have a social stigma attached to it. Studies have proven this to be true, even in the more conservative Middle Eastern countries [11-12].

The present study had 45.31% respondents with a medical background, primarily medical and dental undergraduate students. The number of respondents with a medical background harboring the habit of hookah smoking themselves was significantly lower than those with a non-medical background (28.63% in respondents with a medical background as compared to 63.42% in respondents with a non-medical background, P < 0.05). However, the percentage of respondents who believed cigarettes to be more harmful were not very different in either group (75.11% respondents with a medical background compared to 80.15% respondents with a non-medical background). In fact, the group of respondents with a medical background who smoked hookah had a stronger belief that cigarettes were more injurious than the hookah, although the difference was not statistically significant (93.44% hookah smokers with a medical background compared to 88.95% in hookah smokers with a non-medical background). This pattern of perception of hookah smokers that cigarettes are more harmful has been observed in many previous studies [13-15], although there are some studies that have reported otherwise [16]. It has been proven repeatedly that hookah smoking is more harmful than cigarette smoking because the amount of carbon monoxide and smoke volume is significantly higher. Cobb, et al. reported that peak nicotine exposure was similar in a single session of hookah and cigarette but the carboxyhemoglobin concentration was 3.75 times more and the inhaled smoke volume was 56 times more in a single session of hookah smoking [17]. Aboaziza, et al. reported similar findings [18]. There is a widely held belief that hookah smoking is safer compared to cigarettes because the smoke from the head of the hookah passes through water before coming to the mouthpiece. This is based on the pretext that nicotine is water-soluble. Shihadeh reported that, although some amount of nicotine is removed by passage of smoke from the water, a significant amount still reaches the mouthpiece [19]. Shafagoh, et al. reported that only one-twentieth of the nicotine is trapped in the water of the hookah [20].

In the present study, the majority of the respondents were ignorant of the presence of tobacco in the hookah they smoked. Surprisingly, respondents with a medical background were more ignorant than those with a non-medical background, and the difference was statistically significant (86.88% in respondents with a medical background as compared to 69.32% of respondents). It is a widespread belief that the so-called “herbal” hookah products are considerably safer than the tobacco-containing hookah products. This belief gives a false sense of security to the hookah smoker that they are not indulging in a harmful habit, which is far from the truth. Shihadeh, et al. reported that apart from the lower nicotine content of the “herbal” hookah, there was no significant difference in the amount of carbon monoxide, tar, various carcinogens, and volatile aldehydes between the “herbal” and regular hookah [21].

The average duration of the hookah smoking habit was 3.52 years (95% CI of 3.21 to 3.82 years) while the frequency of use per month was 1.946 (95% CI 1.799 to 2.093) times per month. The average time of a “session” of hookah smoking was 58.90 minutes (95% CI of 54.42 to 63.37). Maziak in 2008 reported that a single session of hookah smoking delivers approximately 50 times the amount of polycyclic aromatic hydrocarbons (which are potential carcinogens) compared to a single cigarette [5].

In the present study, 97.32% respondents reported that they did not smoke hookah alone. This meant that the majority of the time hookah smoking was undertaken as a group activity. These results are consistent with other studies [16, 22]. When undertaken as a group activity, hookah smoking is even more harmful as the time and the amount of passive smoke inhaled increases considerably.

The number of participants who positively responded to smoking hookah and something else was 58.04% while the respondents who smoked something else but not hookah were 30.48%. This pattern of people taking up more than one form of tobacco consumption has been observed in numerous studies [10, 23]. Poyrazoğlu, et al. reported the prevalence of water pipe smoking to be nine times greater in students who smoked a cigarette [16].

## Conclusions

In the present study, the majority of the respondents with a medical background did not foster the hookah smoking habit themselves. However, their perception of hookah smoking and its associated health hazards was not significantly different from people with a non-medical background. Physicians and dental surgeons are at the forefront of the crusade against tobacco usage. They can be successful in the same only when they do not nurture the habit themselves and have a fair amount of accurate knowledge about the ill effects of tobacco usage in all forms. This knowledge should be made a part of the formal teaching curriculum for both medical and dental undergraduate students.    
